# Effect of Ewe Nutritional Restriction During Gestation on the Number of Sertoli Cells in the Offspring: A Meta‐Analysis

**DOI:** 10.1111/rda.70185

**Published:** 2026-02-10

**Authors:** Mario A. Espinosa‐Martínez, Héctor Jiménez‐Severiano, Luis J. Montiel‐Olguín

**Affiliations:** ^1^ CENID Fisiología y Mejoramiento Animal‐INIFAP, Colón Querétaro Mexico

**Keywords:** foetal programming, prenatal stress, testicular development

## Abstract

Maternal nutrition during gestation critically influences foetal reproductive programming. To clarify inconsistent evidence, a meta‐analysis was performed to assess the effect of maternal nutritional restriction on the number of Sertoli cells in ovine offspring. A systematic review in PubMed, Google Scholar, Scopus and Science Direct identified three studies (four comparisons; *n* = 52). Standardised mean differences (Hedges' *g*) were calculated using a random‐effects model. The pooled effect was significant (*g* = −0.95, 95% CI: −1.49 to −0.41; *p* < 0.001), with no heterogeneity (*I* ^2^ = 0%) or publication bias (Egger's test *p* = 0.616). In conclusion, maternal undernutrition reduces Sertoli cell numbers by approximately one standard deviation.

## 1 Introduction

Maternal nutrition during gestation is crucial for foetal development and reproductive programming. Undernutrition can cause lasting alterations in sensitive tissues, including the testes (Bielli et al. 2002; Jaquiery et al. 2012), potentially compromising male fertility. Although several studies link maternal restriction to reduced Sertoli cell numbers, results remain inconsistent (Hoffman et al. 2018; Rae et al. 2002; Kotsampasi et al. 2009). This study performed a meta‐analysis to quantify this effect in sheep offspring.

## 2 Material and Methods

### 2.1 Ethics Statement

This study did not involve live animals; therefore, ethical approval was not required.

### 2.2 Literature Search

Literature search was conducted in PubMed, Google Scholar, Scopus and Science Direct using terms related to maternal undernutrition, gestation, testicular development and sheep. Boolean operators combined descriptors such as ‘maternal undernutrition’, ‘gestation’, ‘Sertoli cells’, and ‘sheep’, applied to title/abstract and MeSH fields. Equivalent strategies were used in the other databases. No date restrictions were applied. The search was conducted in March 2025.

### 2.3 Inclusion and Exclusion Criteria

Experimental studies involving pregnant ewes subjected to dietary restriction from day 0 of gestation were included if they reported the number of Sertoli cells in the progeny. Only peer‐reviewed articles in English were considered. Studies without control groups, reviews, case reports or theses were excluded. Two reviewers independently screened titles and abstracts, and full texts were assessed for eligibility; no discrepancies occurred between reviewers.

### 2.4 Studies Selected for the Meta‐Analysis

**FIGURE 1:**
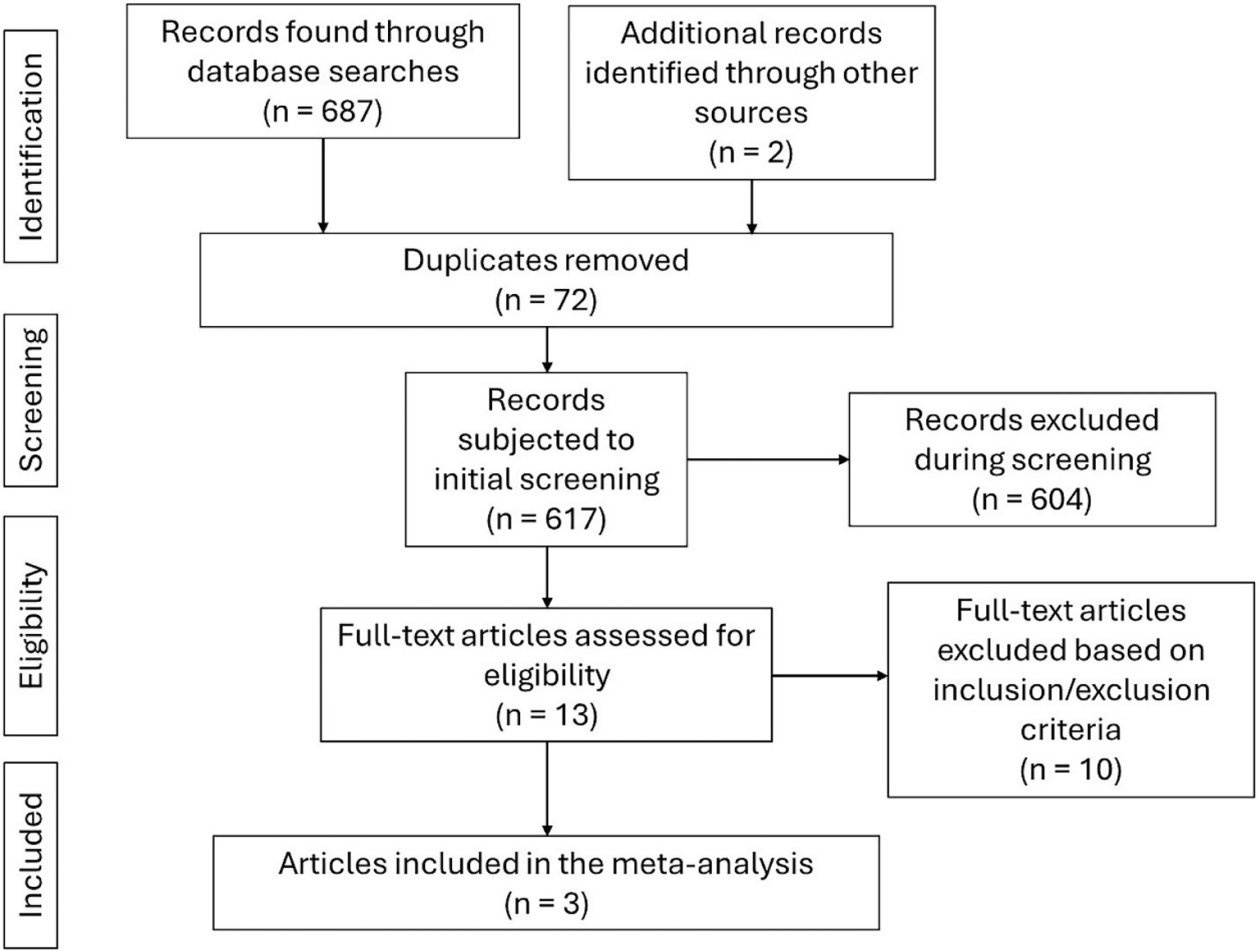
Flow diagram of the identification, screening and selection process for studies included in the meta‐analysis.

Figure 1 presents the flow diagram of study selection. After removing duplicates, 13 studies were reviewed in full. Andrade et al. (2013) and Da Silva et al. (2001) were excluded because Sertoli cells were measured in foetuses, while Mossa et al. (2018) was excluded as diets remained isoenergetic despite changes in starch and sugar ratios. Deligeorgis et al. (1996), Jaquiery et al. (2012), McGovern et al. (2015), Rae et al. (2002), Smith et al. (2010), Ungerfeld et al. (2024) and Zhu et al. (2023) were excluded for not reporting Sertoli cell numbers. Kotsampasi et al. (2009) was included with its control and two experimental groups analysed independently, as both met the inclusion criteria. In total, four studies fulfilled the eligibility requirements and were included in the meta‐analysis.

### 2.5 Statistical Analysis

To account for variation in the methodological approaches used to quantify Sertoli cells across studies (Table 1), effect sizes were computed as standardised mean differences (Hedges' *g*) within a random‐effects model (DerSimonian and Laird 2015). Publication bias was tested using funnel plots and Egger's test, and heterogeneity via *Q* and *I* ^2^ statistics. Analyses were performed in Python (v.3.11.11) with pandas, numpy, matplotlib and statsmodels libraries.

**TABLE 1:** Studies included in the meta‐analysis on the effect of maternal nutritional restriction during gestation on the number of Sertoli cells in the offspring.

Study	Breed	Restricted group	Age at measurement	Number of Sertoli cells, mean ± standard error
Bielli et al. (2002)	Merino	Ewes fed 70% of maintenance energy requirements from week 10 of gestation until lambing	2 days old	Control: 42.98 ± 2.45 × 10^8^ Sertoli cells per testis (*n* = 12) Restricted: 34.52 ± 2.03 × 10^8^ Sertoli cells per testis (*n* = 13)
Hoffman et al. (2018)	Hampshire Down	Ewes fed 50% of energy requirements from ~day 100 of gestation until lambing	60 days old	Control: 151.16 ± 75.23 Sertoli cells per microscopic field at 100× (*n* = 4) Restricted: 91.95 ± 49.89 Sertoli cells per microscopic field at 100× (*n* = 4)
Kotsampasi et al. (2009)—R1	Chios	Ewes fed 50% of nutritional requirements from day 0 to day 30 of gestation	10 months old	Control: 12.17 ± 1.14 Sertoli cells per seminiferous tubule (*n* = 7) Restricted: 9.85 ± 0.75 Sertoli cells per seminiferous tubule (*n* = 7)
Kotsampasi et al. (2009)—R2	Chios	Ewes fed 50% of nutritional requirements from day 31 to day 100 of gestation	10 months old	Control: 12.17 ± 1.14 Sertoli cells per seminiferous tubule (*n* = 7) Restricted: 8.51 ± 0.73 Sertoli cells per seminiferous tubule (*n* = 5)

Abbreviations: *n*, sample size; R1, restricted group 1; R2, restricted group 2.

## 3 Results

**FIGURE 2:**
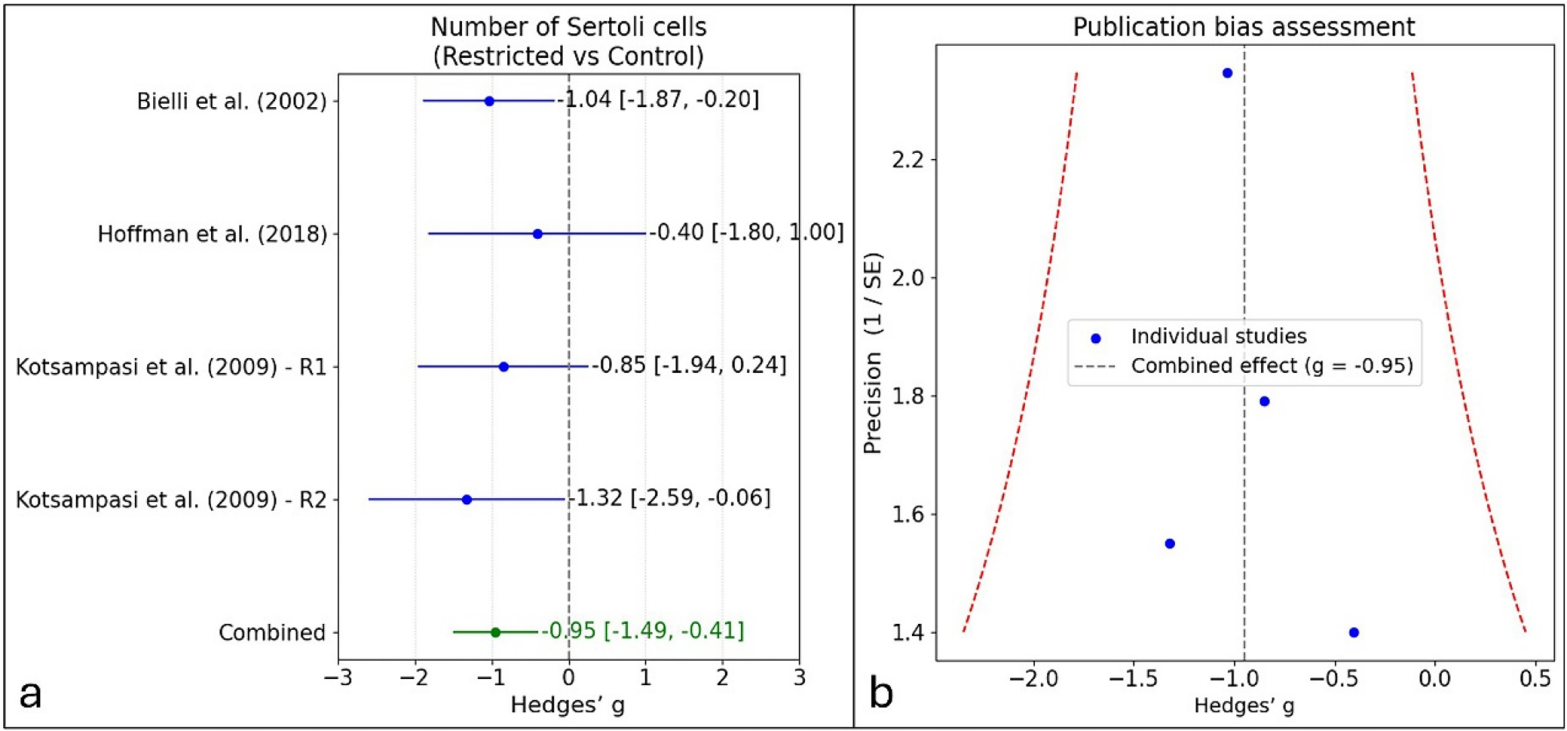
Effect of maternal nutritional restriction during gestation on offspring Sertoli cell number. (a) Forest plot showing individual and pooled effect sizes (Hedges' *g*, 95% CI) for restricted vs. control diets; negative values indicate fewer Sertoli cells in restricted groups. (b) Funnel plot assessing publication bias.

Table 1 summarises the studies included in the meta‐analysis (*n* = 52). Effect sizes (Hedges' *g*) were as follows: Bielli et al. (2002) = −1.04, Hoffman et al. (2018) = −0.40, Kotsampasi et al. (2009) R1 = −0.85 and R2 = −1.32. The pooled random‐effects estimate was *g* = −0.95 (95% CI: −1.49 to −0.41; *p* < 0.001), favouring controls (Figure 2a). No publication bias was detected (Egger's *p* = 0.616), and heterogeneity was negligible (*Q* = 0.99, df = 3, *p* = 0.803; *I* ^2^ = 0%), indicating consistent results across studies (Figure 2b).

## 4 Discussion

Although based on only three articles and four studies, this meta‐analysis remains scientifically valid. Herbison et al. (2011) demonstrated that analyses with as few as three to five studies often yield stable pooled estimates.

Maternal undernutrition during gestation reduced offspring Sertoli cell numbers; although a previous meta‐analysis found no significant effect (Asmad et al. 2012), inclusion of foetal measurements may have diluted the estimate, whereas the present analysis—restricted to postnatal animals and incorporating an additional study (Hoffman et al. 2018)—likely improved sensitivity and statistical power to detect the true effect.

Despite differences in breed, restriction duration and age at evaluation (Bielli et al. 2002; Hoffman et al. 2018; Kotsampasi et al. 2009), all studies reported decreases when restriction began after day 30 of gestation. Only the R1 group of Kotsampasi et al. (2009), restricted from days 0 to 30, showed no change, indicating that the timing of undernutrition is critical (Roca Fraga et al. 2018). Sertoli cell proliferation intensifies after the first month of gestation, explaining the lack of effect from early restriction.

The combined evidence supports that maternal undernutrition reduces Sertoli cell numbers. Despite methodological variation among studies, meta‐analysis allows integration of consistent findings into a robust conclusion. However, the limited dataset and absence of fertility outcomes constrain the evaluation of long‐term reproductive effects, requiring confirmation in future studies.

## 5 Conclusion

Maternal undernutrition during gestation decreases offspring Sertoli cell numbers by about one standard deviation.

## Author Contributions

M.A.E.‐M. investigation, and data curation. H.J.‐S. contributed to funding acquisition and supervised the project. L.J.M.‐O. conceptualization, investigation, formal analysis and prepared the draft. All authors approved the final version.

## Funding

This work was supported by the Instituto Nacional de Investigaciones Forestales, Agrícolas y Pecuarias (12331636360).

## Conflicts of Interest

The authors declare no conflicts of interest.

## Data Availability

All data analysed were obtained from previously published and referenced studies.

## References

[rda70185-bib-0001] Andrade, L. P. , S. M. Rhind , M. T. Rae , C. E. Kyle , J. Jowett , and R. G. Lea . 2013. “Maternal Undernutrition Does Not Alter Sertoli Cell Numbers or the Expression of Key Developmental Markers in the Mid‐Gestation Ovine Fetal Testis.” Journal of Negative Results in Biomedicine 12: 1–8. 10.1186/1477-5751-12-2. 23295129 PMC3584724

[rda70185-bib-0002] Asmad, K. , S. Nakagawa , N. Lopez‐Villalobos , P. R. Kenyon , S. J. Pain , and H. T. Blair . 2012. “Effects of Maternal Nutrition During Pregnancy on the Growth and Reproductive Development of Male Sheep: A Meta‐Analysis.” Proceedings of the New Zealand Society of Animal Production 72: 51–57. https://www.nzsap.org/system/files/proceedings/2012/ab12012.pdf.

[rda70185-bib-0003] Bielli, A. , R. Pérez , G. Pedrana , et al. 2002. “Low Maternal Nutrition During Pregnancy Reduces the Number of Sertoli Cells in the Newborn Lamb.” Reproduction, Fertility and Development 14, no. 6: 333–337. 10.1071/RD02046. 12467358

[rda70185-bib-0004] Da Silva, P. , R. P. Aitken , S. M. Rhind , P. A. Racey , and J. M. Wallace . 2001. “Influence of Placentally Mediated Fetal Growth Restriction on the Onset of Puberty in Male and Female Lambs.” Reproduction 122, no. 3: 375–383. 10.1530/rep.0.1220375. 11597303

[rda70185-bib-0005] Deligeorgis, S. G. , S. Chadio , and J. Menegatos . 1996. “Pituitary Responsiveness to GnRH in Lambs Undernourished During Fetal Life.” Animal Reproduction Science 43, no. 2–3: 113–121. 10.1016/0378-4320(96)01471-6.

[rda70185-bib-0006] DerSimonian, R. , and N. Laird . 2015. “Meta‐Analysis in Clinical Trials Revisited.” Contemporary Clinical Trials 45: 139–145. 10.1016/j.cct.2015.09.002. 26343745 PMC4639420

[rda70185-bib-0007] Herbison, P. , J. Hay‐Smith , and W. J. Gillespie . 2011. “Meta‐Analyses of Small Numbers of Trials Often Agree With Longer‐Term Results.” Journal of Clinical Epidemiology 64, no. 2: 145–153. 10.1016/j.jclinepi.2010.02.017. 20609563

[rda70185-bib-0008] Hoffman, F. , E. Boretto , S. Vitale , et al. 2018. “Maternal Nutritional Restriction During Late Gestation Impairs Development of the Reproductive Organs in Both Male and Female Lambs.” Theriogenology 108: 331–338. 10.1016/j.theriogenology.2017.12.23. 29288977

[rda70185-bib-0009] Jaquiery, A. L. , M. H. Oliver , M. Honeyfield‐Ross , J. E. Harding , and F. H. Bloomfield . 2012. “Periconceptional Undernutrition in Sheep Affects Adult Phenotype Only in Males.” Journal of Nutrition and Metabolism 2012, no. 1: 123610. 10.1155/2012/123610. 23091706 PMC3468125

[rda70185-bib-0010] Kotsampasi, B. , C. Balaskas , G. Papadomichelakis , and S. E. Chadio . 2009. “Reduced Sertoli Cell Number and Altered Pituitary Responsiveness in Male Lambs Undernourished In Utero.” Animal Reproduction Science 114, no. 1–3: 135–147. 10.1016/j.anireprosci.2008.08.017. 18814977

[rda70185-bib-0011] McGovern, F. M. , F. P. Campion , T. Sweeney , S. Fair , S. Lott , and T. M. Boland . 2015. “Altering Ewe Nutrition in Late Gestation: II. The Impact on Fetal Development and Offspring Performance.” Journal of Animal Science 93, no. 10: 4873–4882. 10.2527/jas.2015-9020. 26523580

[rda70185-bib-0012] Mossa, F. , D. Bebbere , A. Ledda , et al. 2018. “Testicular Development in Male Lambs Prenatally Exposed to a High‐Starch Diet.” Molecular Reproduction and Development 85, no. 5: 406–416. 10.1002/mrd.22974. 29542837

[rda70185-bib-0013] Rae, M. T. , C. E. Kyle , D. W. Miller , A. J. Hammond , A. N. Brooks , and S. M. Rhind . 2002. “The Effects of Undernutrition, In Utero, on Reproductive Function in Adult Male and Female Sheep.” Animal Reproduction Science 72, no. 1–2: 63–71. 10.1016/S0378-4320(02)00068-4. 12106966

[rda70185-bib-0014] Roca Fraga, F. J. , M. Lagisz , S. Nakagawa , N. Lopez‐Villalobos , H. T. Blair , and P. R. Kenyon . 2018. “Meta‐Analysis of Lamb Birth Weight as Influenced by Pregnancy Nutrition of Multiparous Ewes.” Journal of Animal Science 96, no. 5: 1962–1977. 10.1093/jas/sky072. 29506123 PMC6140851

[rda70185-bib-0015] Smith, N. A. , F. M. McAuliffe , K. Quinn , P. Lonergan , and A. C. O. Evans . 2010. “The Negative Effects of a Short Period of Maternal Undernutrition at Conception on the Glucose–Insulin System of Offspring in Sheep.” Animal Reproduction Science 121, no. 1–2: 94–100. 10.1016/j.anireprosci.2010.05.001. 20537471

[rda70185-bib-0016] Ungerfeld, R. , F. Sales , A. Freitas‐de‐Melo , and V. H. Parraguez . 2024. “Reproductive Development of Male Lambs Born to Undernourished Mothers Supplemented or Not With Concentrate During Gestation.” Livestock Science 281: 105428. 10.1016/j.livsci.2024.205428.

[rda70185-bib-0017] Zhu, L. , N. Tillquist , G. Scatolin , et al. 2023. “Maternal Restricted‐ and Over‐Feeding During Gestation Perturb Offspring Sperm Epigenome in Sheep.” Reproduction 166, no. 5: 311–322. 10.1530/REP-23-0074. 37647207 PMC10962644

